# Serological identification of fast progressors of structural damage with rheumatoid arthritis

**DOI:** 10.1186/ar4266

**Published:** 2013-08-14

**Authors:** Anne Sofie Siebuhr, Anne C Bay-Jensen, Diana J Leeming, Adam Plat, Inger Byrjalsen, Claus Christiansen, Désirée van de Heijde, Morten A Karsdal

**Affiliations:** 1 Nordic Bioscience, Herlev Hovedgade, DK-2730 Herlev, Denmark; 2 Roche Pharmaceuticals, Shire Park, 1 Falcon Way, Welwyn Garden City, Hertfordshire AL7 1TW, UK; 3 Centre of Clinical and Basic Research, Telegrafvej 4, DK-2750 Ballerup, Denmark; 4 Department of Rheumatology, Leiden University, Albinusdreef 2, NL-2333 Leiden, The Netherlands

**Keywords:** Structural progression, Connective tissue degradation, Biochemical marke, Identification of patients

## Abstract

**Introduction:**

Rheumatoid arthritis (RA) patients with structural progression are in most need of immediate treatment to maintain tissue integrity. The serum protein fingerprint, type I collagen degradation mediated by matrix metalloproteinases (MMP)-cleavage (C1M), is a biomarker of tissue destruction. We investigated whether baseline serum C1M levels could identify structural progressors and if the biomarker levels changed during anti-inflammatory treatment with tocilizumab (TCZ).

**Methods:**

The LITHE-biomarker study (NCT00106535, *n *= 585) was a one-year phase III, double-blind, placebo (PBO)-controlled, parallel group study of TCZ 4 or 8 mg/kg every four weeks, in RA patients on stable doses of methotrexate (MTX). Spearman's ranked correlation was used to assess the correlation between baseline C1M levels and structural progression at baseline and at weeks 24 and 52. Multivariate regression was performed for delta structural progression. Change in C1M levels were studied as a function of time and treatment.

**Results:**

At baseline, C1M was significantly correlated to C-reactive protein (*P *<0.0001), visual analog scale pain (*P *<0.0001), disease activity score28-erythrocyte sedimentation rate (DAS28-ESR) (*P *<0.0001), joint space narrowing (JSN) (*P *= 0.0056) and modified total Sharp score (mTSS) (*P *= 0.0006). Baseline C1M was significantly correlated with delta-JSN at Week 24 (R^2 ^= 0.09, *P *= 0.0001) and at Week 52 (R^2 ^= 0.27, *P *<0.0001), and with delta-mTSS at 24 weeks (R^2 ^= 0.006, *P *= 0.0015) and strongly at 52 weeks (R^2 ^= 0.013, *P *<0.0001) in the PBO group. C1M levels were dose-dependently reduced in the TCZ + MTX group.

**Conclusions:**

Baseline C1M levels correlated with worsening joint structure over one year. Serum C1M levels may enable identification of those RA patients that are in most need of aggressive treatment

**Trial registration:**

ClinicalTrials.gov: NCT00106535

## Introduction

Rheumatoid arthritis (RA) is a chronic autoimmune disease characterized by inflammation and massive tissue destruction in multiple synovial joints. Aggressive treatment for patients whose disease is rapidly progressing (progressors) may preserve joint function. However, it is very difficult using standard technologies to identify progressors. Identification of progressors is also important as most anti-inflammatory treatments are associated with side effects emphasizing that only those who are in most need of aggressive treatment (that is, progressors) should be appropriately treated, which may improve the benefit-risk ratio of these therapies.

In RA, the periarticular bone and cartilage undergo major destructive changes, whereas the altered turnover of the synovial membrane results in an expansion of the synovial membrane. The extracellular matrix (ECM) is the most prominent component of these tissues and the most abundant ECM protein is type I collagen. However, type II collagen is the most abundant protein in cartilage and the most investigated collagen with arthritides. Destruction of the ECM is mediated by enzymatic cleavage by different proteases, though predominantly by matrix metalloproteinases (MMPs). MMPs have been shown to be highly up-regulated with RA [1-3] and the tissue destruction with specific MMPs elevated with disease is a central player in inflammatory disease, such as RA [3,4]. The action of MMPs on ECM results in the release of small protein 'fingerprints' into the circulation, which may be used as markers of tissue destruction [5,6]. It seems reasonable to hypothesize that connective tissue destruction mediated by MMPs could be associated with disease progression in RA.

**Figure 1:**
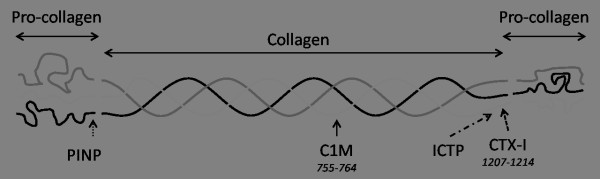
**Biochemical markers of type I collagen**. PINP, Pro-collagen type I N-terminal pro-peptide; C1M, MMP-degraded type I collagen. The C1M peptide is located at amino acid position 755 to 764 in the mature collagen strands. ICTP, Pyridinoline cross-linked carboxyterminal telopeptide of type I collagen. CTX-I, cross-linked c-telo-peptide of type I collagen at amino acid position 1207 to 1214.

Type I collagen can be degraded by several proteinases and in the process yields various products, namely CTX-I, ICTP, PINP and C1M (Figure 1). CTX-I is a measurement of Cathepsin K-mediated destruction of type I collagen and is the standard measure for bone resorption [7]. ICTP is a triple cross-linked carboxyterminal telopeptide of type I collagen generated by MMPs and destroyed by Cathepsin K and, therefore, not related to bone type I collagen [7,8]. Lastly, PINP is widely used as a measure of type I collagen formation in bone [9-12].

We recently developed and validated an enzyme-linked immunosorbent assay (ELISA) using a monoclonal antibody detecting a degradation fragment of the helical domain of type I collagen mediated by MMP-cleavage and destroyed by Cathepsin K (C1M) [13]. This ELISA does not recognize intact type I collagen, other collagens, or the products of other proteinase-mediated collagen degradation. C1M is a product of MMP-2, -9 and -13 cleavage of type I collagen, but not MMP-3, -8 or Cathepsin K cleavage. Therefore, C1M is not a biomarker of hard tissue (that is, bone), but a biomarker of soft tissue destruction, due to the destruction of the fragment by Cathepsin K. Therefore, it represents another pool of type I collagen degradation than PINP, CTX-I and ICTP, namely, exclusively MMP-mediated connective tissue destruction [13].

The aim of the present study was to investigate whether MMP-mediated type I collagen destruction, measured by serum levels of C1M, could be a novel biochemical marker for identification of progressors in RA. We investigated whether baseline levels of serum C1M were correlated with structural status and progression of RA, by measuring change in joint space narrowing, total Sharp score and erosion, in the placebo group of the biomarker study of the LITHE study [14]. We, furthermore, investigated whether serum C1M levels were altered following treatment of RA with an anti-IL-6R monoclonal antibody, tocilizumab (TCZ), in combination with methotrexate (MTX) in the biomarker study in the LITHE study [14,15]. Lastly, we investigated if the change in C1M at four weeks was related to change in one year radiographic measures and if including the standard biomarker (C-reactive protein, CRP) of RA increased the predictive value of C1M.

## Material and methods

### LITHE study

The LITHE Biomarker study formed part of the LITHE study (ClinicalTrials.goc: NCT00106535), which was a two-year phase III, double-blind, placebo-controlled, parallel group study of TCZ (4 mg/kg or 8 mg/kg every four weeks) + MTX (10 to 25 mg/week) in patients with moderate to severe active RA with an inadequate response to MTX. RA was diagnosed according to the American College of Rheumatology criteria [16], and had to have lasted for six or more months before entering the study. Moreover, the patients had to have radiographically confirmed joint erosion.

The LITHE Biomarker study included 585 patients followed for the first 52 weeks from baseline. Biomarker patients were equally distributed among the treatment groups; placebo (PBO; *n *= 199), 4 mg/kg TCZ (TCZ4; *n *= 200) and 8 mg/kg TCZ (TCZ8; *n *= 186). Stable non-steroidal anti-inflammatory drugs and corticosteroid (≤10 mg/day prednisone or equivalent) doses were continued throughout the study. Every 4 weeks, patients received an infusion of TCZ 4 mg/kg, 8 mg/kg, or placebo for a total of up to 13 infusions in 52 weeks. Serum for biomarker research was collected following overnight fasting at baseline, at weeks 4, 16, 24 and 52 and non-fasted at Week 2. All samples were stored below -70°C until assay.

**Figure 2:**
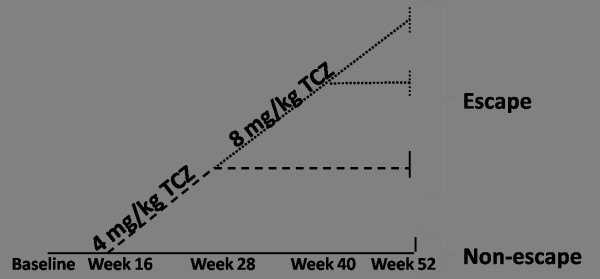
**Study design of the PBO + MTX group (*n *= 199) in the LITHE biomarker study**. Patients with <20% improvement (escape patients) at Week 16 were given 4 mg/kg TCZ + MTX every four weeks from Week 16. At Week 28 PBO + MTX patients who had received 4 mg/kg TCZ + MTX were evaluated and patients with <20% were given 8 mg/kg TCZ + MTX every four weeks. Responders to TCZ (>20% improvement) remained on 4 mg/kg TCZ + MTX for the rest of the study. If patients who went on to 8 mg/kg TCZ + MTX did not respond after three doses, they were excluded from the remainder of the study, but were included in the Week 24 analysis. Dashed line: First rescue therapy. Dotted line: Second stage rescue therapy. MTX, methotrexate; PBO, placebo; TCZ, tocilizumab

Patients who failed to respond to treatment during the study, that is, experienced ≤20% improvement from baseline in both swollen (SJC) and tender joint counts (TJC) at Week 16, could receive blinded rescue therapy from Week 16, and subsequently, 12 weeks later (at Week 28) if there was still ≤20% improvement. First-step rescue patients receiving PBO + MTX switched at Week 16 to TCZ 4 mg/kg + MTX. Patients receiving 4 mg/kg TCZ + MTX switched to 8 mg/kg + MTX and patients on 8 mg/kg + MTX discontinued treatment. Second-step rescue at Week 28 consisted of TCZ 8 mg/kg + MTX, which were offered through to Week 52 if inadequate response persisted after three doses of first-step rescue therapy. Patients who did not respond after three doses of second-step rescue discontinued treatment. The patients from the PBO + MTX group, who received rescue therapy, were designated escapers. Half of the patients receiving PBO + MTX at baseline were receiving first step rescue therapy at Week 52. Only six patients were on second step rescue therapy at Week 52 (Figure 2).

Radiographs of hands and feet were obtained at baseline, Week 24 and Week 52. Radiographs were assessed with joint space narrowing (JSN), the total Genant-modified Sharp scoring system (mTSS) [17] and erosion by two independent readers, who were blinded to treatment assignment, chronological order of radiographs and patients' clinical responses. The average score of the two readers was used in the analysis. The visual analog scale of pain (VAS) and disease activity score-28-erythrocyte sedimentation rate (DAS28-ESR) were assessed at baseline, and at weeks 24 and 52. The self-reported general health was assessed by the Health Assessment Questionnaire (HAQ) at baseline, weeks 24 and 52.

The LITHE study was approved by ethical committees at each participating institution [14]. All patients in the LITHE study provided written informed consent. According to Danish law, it is not required to get ethical approval when measuring biochemical markers in previously collected samples, hence, there was no ethical approval for this particular study of C1M in the LITHE serum samples. The study was conducted according to the Principal of Good Clinical Practice and according to the Declaration of Helsinki.

### Biochemical markers

CRP was quantified in patients at baseline, 24 and 52 weeks. C1M was measured in samples taken at baseline and at weeks 2, 4, 16, 24 and 52 using the newly developed C1M ELISA assay (11), which is based on a monoclonal antibody recognizing the fragment GSPGKDGVRG at position 764 in the mature type I collagen.

### Statistics

Summary statistics were used for general demographics, baseline RA characteristics and baseline American College of Rheumatology demographics. Spearman's ranked correlation was conducted on baseline level of serum C1M, age, body mass index (BMI), disease duration, VAS pain, DAS28-ESR, HAQ, JSN and mTSS. The same correlation test was applied to compare baseline C1M with change in CRP, VAS pain, HAQ, DAS28-ESR, JSN and mTSS at weeks 24 and 52.

Multiple regression analysis was performed on log-transformed data for delta structural progression (JSN, mTSS and erosion score) and C1M, with the cofounders age, BMI, disease duration, baseline CRP and baseline structural status.

Changes from baseline in serum C1M levels were studied as a function of time and treatment. Differences between the levels of serum C1M at each time point were compared by two-way ANOVA. The level of change from baseline in C1M for escape patients and non-escape patients in the PBO groups was assessed by two-way ANOVA.

To test if CRP increased the predictive value of C1M to predict progression, high and low levels of C1M and CRP were determined as higher and lower the respective median. This provided four groups: 1: High C1M and high CRP, 2: High C1M and low CRP, 3: Low C1M and high CRP and 4: low C1M and low CRP. Odds ratio for structural progression (JSN and mTSS) were assessed between group 4 and the other groups. The odds ratio for C1M alone was furthermore determined.

## Results

### Patient demographics at baseline

A total of 585 patients were included at baseline and baseline characteristics are listed in Table 1. Of the 199 patients in the PBO + MTX group 109 patients received rescue at Week 16. The percentage of women in the study was 83%. At baseline, the mean age of the biomarker study population was 51.6 years (SD: 12.6), mean BMI was 28.1 kg/m^2 ^(SD: 7.4) and mean disease duration was 9.3 years (SD: 8.2). The mean HAQ score was 1.54 (SD: 0.61) and mean VAS pain was 54.8 (SD: 21.3). Mean DAS28-ESR was 6.5 (SD: 0.98), mean JSN was 11.6 (SD: 15.7), mean mTSS was 27.9 (SD: 32.3) and mean erosion score was 16.6 (SD: 16.3). The mean CRP was 2.15 mg/dL (SD: 2.52) and C1M was 109 nmol/L (SD: 72).

**Table 1:** Baseline characteristics of the Biomarker Study population

N	585	
% Women	83%	
	*Mean*	*SD*
Age (years)	52.2	12.3
BMI (kg/m^2^)	27.4	6.1
Disease duration (years)	9.4	8.1
Disease parameters		
HAQ	1.51	0.62
VAS pain	54.4	22.1
TJC (n)	28	16
SJC (n)	16	9
DAS28-ESR	6.5	0.93
JSN	11.6	14.4
mTSS	28.4	29.6
ERN	16.6	16.3
Serum biomarkers		
CRP (mg/L)	2.13	2.57
C1M (nmol/L)	109	72.0

BMI, Body mass index; CRP, C-reactive protein; DAS28-ESR, Disease activity score 28-ESR; ERN, Erosion score; HAQ, Health assessment questionnaire; JSN, joint space narrowing; mTSS, modified total Sharp score; SJC, Swollen joint count; TJC, Tender joint count; VAS, Visual analog scale of pain

### Univariate analysis

Baseline C1M was not correlated to age, BMI or disease duration (Table 2). Baseline C1M was highly correlated to delta serum CRP in all groups at both weeks 24 and 52 (Table 2). At baseline, C1M was positively correlated to baseline CRP (ρ = 0.8), but at 24 and 52 weeks it was negatively correlated, ρ = -0.31 and ρ = -0.36, respectively. C1M at baseline was significantly correlated to VAS pain (ρ = 21, *P *<0.0001), HAQ (ρ = 0.21, *P *<0.0001), DAS28-ESR (ρ = 0.25, *P *<0.0001) and JSN (ρ = 0.12, *P *= 0.0056). Delta-VAS pain, delta-HAQ, delta-DAS28-ESR and delta JSN were not significantly correlated to baseline C1M at either 24 or 52 weeks in the total PBO + MTX population or the w/o-escape PBO + MTX population.

**Table 2:** Univariate correlation between clinical markers and baseline serum C1M

	Baseline (all)			Δ24 weeks (PBO)			Δ24 weeks (PBO w/o escape)			Δ52 weeks (PBO w/o escape)		
	ρ	*P*	N	ρ	*P*	n	ρ	*P*	n	ρ	*P*	n
**Age**	-0.06	ns	585	-	-	-	-	-	-	-	-	-
**BMI**	0.01	ns	578	-	-	-	-	-	-	-	-	-
**Disdur**	-0.01	ns	584	-	-	-	-	-	-	-	-	-
**CRP**	0.80	<0.0001	584	-0.31	0.001	112	-0.31	0.0023	93	-0.36	0.0007	83
**VAS pain**	0.21	<0.0001	578	-0.08	ns	110	-0.05	ns	91	-0.00	ns	83
**HAQ**	0.21	<0.0001	535	-0.17	ns	98	-0.15	ns	82	-0.11	ns	76
**DAS28**	0.24	<0.0001	573	0.09	ns	108	0.15	ns	89	0.11	ns	77
**JSN**	0.12	0.0056	556	0.08	ns	153	0.16	ns	90	0.21	0.055	84
**mTSS**	0.14	0.0006	556	0.19	0.017	153	0.28	0.0084	90	0.34	0.0017	84
**ERN**	0.15	0.0003	556	0.19	0.019	153	0.13	ns	90	0.21	0.054	84

BMI, Body mass index; DisDur, Disease duration;CRP, C-reactive protein; DAS28-ESR, Disease activity score 28-ESR; HAQ, Health assessment questionnaire; JSN, Joint space narrowing; mTSS, modified total Sharp score; PBO, placebo group; VAS pain, Visual analog scale of pain. Spearman rank correlation coefficient ρ, the *P*-values and n are provided.

At baseline, C1M was significantly correlated to mTSS (ρ = 0.14, *P *= 0.0006). At 24 weeks, when a proportion of the PBO group was receiving rescue treatment, there was a significant correlation between baseline C1M and delta-mTSS (ρ = 0.19, *P *= 0.017). However, this correlation was stronger when patients receiving rescue treatment (*n *= 109) were excluded from the analysis (ρ = 0.28, *P *= 0.0084). At Week 52 there was an even stronger correlation between delta-mTSS and baseline C1M (ρ = 0.34, *P *= 0.0017) in the patients of the PBO + MTX group not receiving rescue therapy.

### Multiple regression analysis

The effect size of C1M was β = 0.34, β = 1.13 and β = 0.79 for the association with the change in structural progression as determined by JSN, mTSS and erosion score at Week 24, respectively. A higher effect size was seen at Week 52 with β = 1.38, β = 3.40 and β = 2.01, respectively. A β-value illustrates the effect of a variable on another variable, meaning that with a little β-value the covariates do not have an effect on the predicting value. When β is large, the variable has big effect on the predicting value.

The significant association increased when CRP was taken into account at both time points for JSN and mTSS. For erosion score, the significant association was eliminated. When the other possible cofounders (age, BMI, disease duration and baseline radiographic score) were included, a slight decrease was detected at both time points with JSN, mTSS and erosion scores (Table 3). Interestingly, the effect of the cofounders on mTSS and erosion score at 24 weeks lowered the β to below the analysis without the cofounders, making the effect of C1M as predicting structural progression less significant.

**Table 3:** Multivariate linear regression analysis of patients in the PBO group, at 24 and 52 weeks.

	Δ24 weeks				Δ52 weeks			
**JSN**	**β**	***P***	**R^2^**	**n**	**β**	***P***	**R^2^**	**n**

**C1M**	0.34	0.013	0.04	153	1.38	0.0015	0.11	85
**C1M adjusted for CRP**	0.74	0.0013	0.06	153	3.91	<0.0001	0.27	85
**C1M adjusted for CRP, age, BMI and disease duration**	0.69	0.0022	0.11	151	3.60	<0.0001	0.30	83
**C1M adjusted for CRP, age, BMI, disease duration and baseline JSN**	0.68	0.0029	0.11	151	3.55	<0.0001	0.31	83
**mTSS**								

**C1M**	1.13	0.0035	0.05	153	3.40	0.0001	0.17	85
**C1M adjusted for CRP**	1.17	0.07	0.04	153	7.13	<0.0001	0.25	85
**C1M adjusted for CRP, age, BMI and disease duration**	0.98	0.11	0.14	151	6.12	<0.0001	0.32	83
**C1M adjusted for CRP, age, BMI, disease duration and baseline mTSS**	0.95	0.13	0.14	151	6.04	<0.0001	0.32	83
**ERN**								

**C1M**	0.79	0.009	0.038	153	2.01	0.0002	0.15	85
**C1M adjusted for CRP**	0.43	0.39	0.037	153	3.22	0.0006	0.17	85
**C1M adjusted for CRP, age, BMI and disease duration**	0.29	0.56	0.12	151	2.52	0.0055	0.25	83
**C1M adjusted for CRP, age, BMI, disease duration and baseline ERN**	0.26	0.59	0.12	151	2.47	0.0066	0.25	83

BMI, Body mass index; C1M, Type I collagen degraded by MMPs; CRP- C-reactive protein; ERN, erosion score; JSN, joint space narrowing; mTSS, modified total Sharp score. The regression coefficient, β, *P*-value and R^2 ^are given. Data were log-transformed before analysis.

The overall regression models showed more significance with higher correlation coefficients at Week 52 than Week 24 (Table 3). The model for changes in JSN was strongly correlated with baseline C1M at both 24 and 52 weeks (*P *= 0.0029 and P <0.0001, respectively). The model for mTSS and erosion at 24 weeks was not correlated with C1M (*P *= 0.13 and *P *= 0.59), but strongly correlated at 52 weeks (*P *<0.0001 and *P *= 0.0066).

### Odds ratio for structural progression

We investigated whether a combination of high baseline C1M (>89.28 nmol/L) and high baseline CRP (>1.42 nmol/L) levels was a stronger predictor of disease progression than C1M alone. Patients with high C1M alone had a significantly higher odds ratio (95% CI) of radiographic progression with both JSN and mTSS than patients with low C1M alone (JSN; 3.15 (1.50 to 6.58), *P *= 0.0023 and mTSS; 2.94 (1.71 to 5.05), *P *= 0.0001) Patients were then categorized according to high or low baseline C1M and high and low CRP determined by the median. Patients having both high C1M and CRP had a significantly higher odds ratio (95% CI) for radiographic progression than patients with both low C1M and CRP (JSN; 3.55, (1.97 to 6.41), *P *<0.0001 and mTSS; 3.6, (1.62 to 8.08), *P *= 0.0018); Table 4). Patients with a combination of high and low biomarkers had only slight but not significantly higher odds for radiographic progression than patients with both low C1M and CRP. Of those patients with both high C1M and CRP, 18% and 34% had radiographic progression by JSN and mTSS, respectively. Only 13% and 6% of patients with both low C1M and CRP had radiographic progression read by JSN and mTSS, respectively. Hence, also testing for CRP level increases the odds ratio from 3.15 and 2.94 to 3.61 and 3.55 for JSN and mTSS, respectively.

**Table 4:** Odds ratios for radiographic progression when combining high and low level of C1M and CRP

C1M/CRP		High/High vs. Low/Low	High/Low vs. Low/Low	Low/High vs. Low/Low	Only C1M
**JSN**	OR	3.61	2.37	1.95	3.15
	95% CI	1.62 to 8.01	0.59 to 9.44	0.39 to 9.77	1.50 to 6.58
	*P*	0.0018	0.22	0.42	0.0023
**mTSS**	OR	3.55	1.79	1.94	2.94
	95% CI	1.97 to 6.41	0.60 to 5.33	0.58 to 6.49	1.71 to 5.05
	*P*	<0.0001	0.30	0.28	0.0001

The high and low levels of the biomarkers were based on the median value of all patients at baseline. C1M, Type I collagen degraded by MMPs; CI, confidence interval; CRP- C-reactive protein; JSN, joint space narrowing; mTSS, modified total Sharp score; OR, odds ratio

### Dose-dependent response

**Figure 3:**
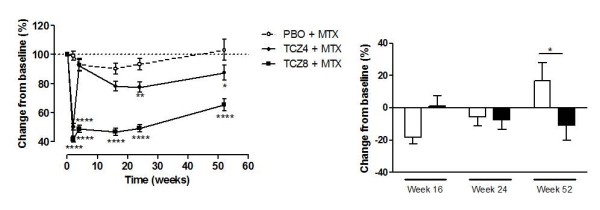
**C1M levels in PBO + MTX or TCZ + MTX treated patients**. **A**: Percentage change in C1M levels from baseline in all patients (including patients, who received TCZ). TCZ4, TCZ 4 mg/kg. TCZ8, TCZ 8 mg/kg. **B**: C1M levels in the subpopulations of the PBO group. Non-escape Week 16 *n *= 86, escape Week 16 *n *= 81. Non-escape Week 24 *n *= 79, escape Week 24 *n *= 78, non-escape Week 52 *n *= 66, escape Week 52 *n *= 56. Values are depicted as mean + SEM. Two-way ANOVA test was used to test for differences. Significant levels: *: *P *<0.05, **: *P *<0.01, ****: *P *<0.0001. C1M, Type I collagen degraded by MMPs; MTX, methotrexate; PBO, placebo; TCZ, tocilizumab

Patients receiving 4 mg/kg TCZ + MTX had a rapid decrease (in the first two weeks) in C1M levels of 49.6% from baseline. However, this effect disappeared at Week 4 and was not significantly different from the C1M levels seen in the PBO + MTX group (Figure 3A). The C1M fragment was detected at a lower level in the 4 mg/kg TCZ + MTX group than the PBO + MTX group at Week 16 (21.6%, *P *>0.05), 24 (22.4%, *P *<0.01), and 52 (12.5%, *P *<0.05). The 8 mg/kg TCZ + MTX group had significantly lower levels of C1M after treatment compared with the PBO + MTX group (Figure 3A). A rapid decrease in C1M was detected at Week 2 (49.2% lower than at baseline, *P *<0.0001), as was seen in the 4 mg/kg TCZ + MTX group. The level of C1M remained at a significantly lower level in the 8 mg/kg TCZ + MTX than in the PBO group throughout the study (*P *<0.0001), although C1M levels increased slightly with time.

To further study the effect of TCZ on C1M levels, the PBO group was separated into escape and non-escape patients (Figure 2). The mean level of C1M at Week 16 was reduced by 18.2% from baseline in the non-escape patients, whereas the mean level was 1% of baseline in the escape patients. The difference in mean level of C1M between escapers and non-escapers was not significant (*P *>0.05). The change from baseline in C1M levels was similar (approximately 6% decrease) in the escape and non-escape patients in the PBO group at Week 24, when the escape patients had been treated with 4 mg/kg TCZ + MTX for eight weeks (Figure 3B). At Week 52 the level of C1M was significantly higher (*P *<0.05) in the non-escape patients than in the escape patients, the latter of which was treated with either 4 or 8 mg/kg TCZ (Figure 3B).

### Change in C1M at 16 weeks correlated to change in radiographic measures at 52 weeks in response to TCZ

**Figure 4:**
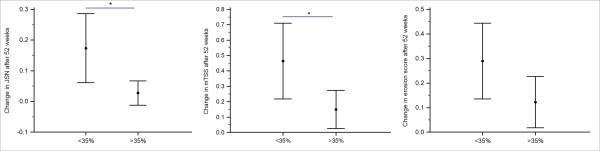
**Change in radiographic measures in big/small change in C1M**. Change in radiographic measures (JSN and mTSS) after 52 weeks in patients receiving TCZ with more or less than 35% change in C1M from baseline to 16 weeks. Values are depicted as mean ± 95% CI. Students' *t*-test was used to test for differences. Significant levels: *: *P *<0.05. C1M, Type I collagen degraded by MMPs; JSN, joint space narrowing; MMPs, matrix metalloproteinases; mTSS, modified total Sharp score; TCZ, tocilizumab

We investigated whether change in C1M after 16 weeks in response to 4 or 8 mg/kg TCZ was related to change in radiographic change after 52 weeks. We separated patients into two groups, those with less than 35% change in C1M from baseline to 16 weeks and those with more than 35% change. A significant difference was found in change in JSN after 52 weeks between the two groups (*P *= 0.035, Figure 4A). A similar difference was seen for mTSS changes (*P *= 0.045, Figure 4B). No difference in erosion was found at either 24 or 52 weeks. There was no change at 24 weeks with JSN and mTSS.

## Discussion

RA is a complex disease in which progression is very heterogeneous and non-continuous, in which flares may be important drivers of severe progression. Selecting the patients who will benefit from a specific treatment would be an advantage in inflammatory arthritis diseases in particular, as most, if not all, anti-inflammatory treatments are associated with severe, but manageable, side effects. In this study we investigated if a novel assay detecting a protein fingerprint of type I collagen degradation could be a novel biochemical marker for identification of progressors in RA. We also investigated the change in serum C1M levels following treatment with TCZ and the predictive value of C1M in combination with CRP. We found that 1) C1M levels were associated with disease progression, 2) C1M levels were correlated with disease activity at baseline, 3) baseline C1M was a significant predictor of structural progression, 4) TCZ dose-dependently inhibited the level of C1M as early as two weeks after initiating treatment, and 5) change in C1M at 16 weeks after treatment initiation was related to the level of radiographic changes over one year. The current findings contribute to the literature and the scientific field by demonstrating that quantifying specific tissue destruction may aid in selecting the RA patients in most need of aggressive treatment.

Serological biochemical markers are receiving increased attention in drug development and for guiding evidence-based patient management decisions [18]. A range of traditional markers may provide valuable in managing RA, such as CRP, ESR, and the interleukins-1 and -6. However, these markers are systemic biomarkers. A newer class of molecular biochemical markers, also assessed by simple ELISA technologies, provides independent and additional information reflecting structural changes within the tissue [5]. These markers - protein fragments - are referred to as protein fingerprints, because they are end results of specific pathological events, proteolytic degradation of, that is, the joint structure [5,6]. The protein fingerprint degradation markers of type I, II and III collagen have been studied and all three have been shown to be descriptive of disease progression and response to anti-inflammatory therapy [19]. In this study, we further show that C1M is highly correlated to CRP levels (Table 2), indicating that type I collagen degradation in RA is related to inflammation. Therefore, exploring C1M in RA will indicate the state of the specific inflammatory structural changes and not the systemic state of the inflammation. Due to the relationship between C1M and CRP, we have not investigated the difference between the biomarkers in predicting progressors of disease.

Interpreting biochemical markers found in serum is associated with many limitations, as several different tissues at different rates may produce, and thus contribute to the total pool of, the molecular marker. Type I collagen is abundant in many tissues throughout the body [5]. Bone in particular may be a prime candidate contributing to the serological C1M pool as it is naturally being constantly renewed. However, C1M has been shown not to be correlated with bone metastases, which have high and very aggressive bone resorption, and, furthermore, not to recognize MMP-3, -8 and Cathepsin K-cleaved type I collagen [13]. Thus, C1M is a biochemical marker with increased accuracy for connective tissue turnover. Other biomarkers of type I collagen degradation have previously been described in the literature in relation to RA. ICTP, a C-terminal degradation fragment of the mature type I collagen, has been shown to be related to the severity of RA [20,21] as well as disease activity [22]. It has been shown to be useful for assessing the effect of potentially disease modifying therapies [23]. ICTP in synovial fluid and serum has been shown to be correlated to radiographic measures of joint destruction [22,24]. However, RA disease duration has shown to be a cofounder in ICTP levels [25]. ICTP has also been described to be correlated to disease activity in reactive arthritis [26]. Another marker of type I collagen, CTX-I, a marker of Cathepsin K-mediated type I collagen degradation reflecting bone resorption, has previously been described to predict disease progression, albeit only in the worst quartile [27,28]. Furthermore, it has been shown in a study of early RA patients that CTX-I at baseline, adjusted for cofounders, was significantly associated with radiographic progression in the RA MRI erosion score and classical radiographs over one year [20]. A drawback of this study, however, was that there was a considerable overlap in CTX-I between progressors and non-progressors. The same group of investigators has shown that the relationship between elevated serum levels of CTX-I and subsequent joint destruction still existed at 10-year follow-up. However, the relationship was weaker [29].

In the assessment of C1M as a marker of structural progression in RA, we have utilized radiographic changes (JSN, mTSS and erosion score) after 24 and 52 weeks. The significance level of the correlation between C1M and radiographic scores was strongest at 52 weeks, especially with mTSS. This is due to the low sensitivity of the radiographic scores to changes in joints over a short period [30]. The low sensitivity led to high variation in scores at 24 weeks. The variation decreased when assessing the radiographic scores at 52 weeks and hence increasing the statistical power. JSN is a visualization of the space between the joints and a reduction in JSN indicates loss of cartilage and meniscal damage, hence only a part on the JSN could is associated with type I collagen, which partly could explain the lack of correlation.

A limitation of this study is the effect of concomitant intake of non-steroidal anti-inflammatory drugs and corticosteroid. These medications could have an influence on the level of C1M, but this contribution cannot be studied in this setting. Especially, prednisolone intake influences the level of inflammation and joint damage [31,32]. However, patients already take stable doses of prednisolone and/or other drugs at the time they enter the study, hence the effect of the drugs might be obliterated in the baseline measurements of C1M. Moreover, the study population of this investigation has long disease duration at baseline and profound joint damage, hence the study population is in an advanced disease stage. It is therefore not a disease population in which a biomarker of progression is needed. A study population with shorter disease duration is more ideal for this type of study. However, the disease progression of these patients is not as profound and an even larger study population would be needed. Our present study aims at identifying progressors of disease by the biomarker by which this study population is adequate, due to the rapid progression.

## Conclusion

We demonstrated that a simple serum biochemical marker measurement is able to predict fast progressors in RA and we also illustrated that C1M could be used as an efficacy marker. It is critical to effectively treat tissue destructive diseases, such as RA, early to suppress inflammation and prevent destruction of the joint.

## Abbreviations

BMI: body mass index; C1M: type I collagen degraded by MMPs; CRP: C-reactive protein; CTX-1: degradation product of c-terminal telopeptides of type I collagen; DAS: Disease Activity Score; ECM: extracellular matrix; ELISA: enzyme-linked immunosorbent assay; ESR: erythrocyte sedimentation rate; HAQ: Health Assessment Questionnaire; JSN: joint space narrowing; MMP: matrix metallo-proteinase; mTSS: modified total Sharp score; MTX: methotrexate; PBO: placebo; RA: rheumatoid arthritis; SJC: swollen joint count; TCZ: tocilizumab; TJC: tender joint count; VAS: visual analog scale

## Competing interests

ASS, ACBJ, DJL, IB and MK are full time employees at and CC and MK hold stock in Nordic Bioscience. Nordic Bioscience is a privately-owned, small- to medium-sized enterprise (SME) partly focused on the development of biomarkers for rheumatic and fibrotic diseases. None of the authors received fees, bonuses or other benefits for the work described in the manuscript. AP and DvdH declare that they have no financial or competing interests.

## Authors' contributions

ASS, ACBJ and MK did the study design. AP provided the samples. Lab-technicians at Nordic Bioscience did the C1M quantification. ASS and ACBJ did the primary data analysis, but all authors were involved in further analysis, interpretation and discussion of data. All authors were involved in the drafting of the article or revising it critically for important intellectual content and all authors approved the final version to be published.
